# Variation in Tdap and Influenza Vaccination Coverage Among Pregnant Women by Insurance Type — Florida, 2016–2018

**DOI:** 10.15585/mmwr.mm6903a4

**Published:** 2020-01-24

**Authors:** Taylor A. Merritt, Sonja A. Rasmussen, Melissa A. Bright, Dikea Roussos-Ross, Shireen Madani Sims, Matthew J. Gurka, Lindsay A. Thompson

**Affiliations:** ^1^University of Florida College of Medicine, Gainesville, Florida; ^2^Department of Pediatrics, University of Florida College of Medicine, ^3^Department of Epidemiology, University of Florida College of Public Health and Health Professions and University of Florida College of Medicine; ^4^Department of Obstetrics and Gynecology, University of Florida; ^5^Department of Health Outcomes and Biomedical Informatics, University of Florida.

Infants are at increased risk for pertussis-associated morbidity and mortality, and pregnant women and their infants are more likely than other patient populations to experience severe influenza-related illness (*1*,*2*). The Advisory Committee on Immunization Practices (ACIP) recommends that all women receive the tetanus toxoid, reduced diphtheria toxoid, and acellular pertussis (Tdap) vaccine during each pregnancy, preferably during the early part of gestational weeks 27–36 (*3*). ACIP also recommends that women who are or might be pregnant during the influenza season receive the inactivated influenza vaccine at any time during pregnancy (*4*). Despite these recommendations, coverage with Tdap and influenza vaccines during pregnancy has been low, with approximately one half of women receiving each vaccine and only one third receiving both, based on a survey during March–April 2019 (*5*). Data obtained through a retrospective chart review of randomly selected pregnant women who delivered at the University of Florida Health Shands Hospital in Gainesville, Florida, from January 1, 2016, to December 31, 2018, were analyzed to assess vaccination coverage by insurance type. Because the Florida Medicaid policy at that time did not cover these vaccines during pregnancy, the hospital system offered Tdap and influenza vaccines at no additional cost to mothers during the immediate postpartum hospital stay. Among 341 women, 68.6% of privately insured and 13.4% with Medicaid received Tdap during pregnancy, and among 316 women, 70.4% of privately insured and 35.6% with Medicaid received influenza vaccine during pregnancy. Many women, especially those with Medicaid, were vaccinated in the immediate postpartum period, when vaccination was available at no cost, increasing Tdap vaccination rates to 79.3% for privately insured and 51.7% for women with Medicaid; influenza vaccination rates rose to 72.0% for privately insured and 43.5% for women with Medicaid. These data suggest that the state Medicaid policy to not cover these vaccines during pregnancy might have significantly reduced coverage among its enrollees.

Pertussis and influenza are associated with substantial morbidity and mortality among infants. Pertussis-related mortality is highest among newborns, who receive the first dose of the diphtheria and tetanus toxoids and acellular pertussis (DTaP) vaccination series at age 2 months (*1*). Influenza vaccine is recommended for all infants aged ≥6 months (*4*). During the period before infants are eligible for vaccination, they rely upon passively acquired transplacental maternal antibodies for protection against these vaccine-preventable diseases. Pregnant women are also at increased risk for severe influenza-associated illness and death (*2*). To provide protection for both mothers and infants, maternal immunization with Tdap is recommended during pregnancy and with influenza vaccine before or during pregnancy, rather than during the postpartum period; vaccination during the postpartum period has been shown to be less effective in preventing infant pertussis (*6*).

Data for this analysis were obtained through a retrospective review of charts of women who delivered a live birth at the University of Florida Health Shands Hospital during 2016 2018. A computer-generated, random selection of 450 women was obtained from the population of 6,949 women with Medicaid or private insurance at the time of their delivery. Among these women, 109 (24.2%) were excluded because they did not meet certain eligibility criteria: 13 (2.9%) were aged <18 years at initial visit, 84 (18.7%) received no prenatal care at University of Florida Health, and 12 (2.6%) delivered at less than 30 weeks’ gestation, thus leaving an initial analytic sample of 341. An additional 25 women for whom the influenza vaccine was not indicated (because of receipt of vaccine just before pregnancy, allergy to a vaccine component, or nonavailability of the vaccine because of late presentation to prenatal care in the brief summer window when vaccine was not available) were excluded from the analysis of influenza vaccination, leaving 316 women in the analysis of influenza vaccination. Women who were not pregnant during influenza season were not specifically excluded; however, a few women were excluded if the vaccine was unavailable when they were seen for prenatal care, effectively excluding women who were seen outside of influenza season.

The primary outcomes assessed were receipt of Tdap and influenza vaccines during pregnancy. The primary predictor was insurance status (Medicaid versus private insurance). Secondary outcomes included receipt of Tdap and influenza vaccines during pregnancy or in the immediate postpartum period (before delivery hospital discharge). Although postpartum vaccination was examined to estimate the number of women who would be responsive to vaccination if financial barriers were removed, other factors might have contributed to this decision. Descriptive statistics for demographic and prenatal care characteristics were calculated overall and by insurance type. Characteristics for which statistically significant differences existed by insurance type were included as covariates in subsequent multivariate analyses.

Unadjusted and adjusted logistic regression models were used to estimate the relationships between insurance type and receipt of Tdap and influenza vaccines during pregnancy* and receipt of Tdap and influenza vaccines in the immediate postpartum period. The models were adjusted for race, age, parity, gestational age at delivery, trimester at initiation of prenatal care, and completion of recommended prenatal initiation studies as a proxy for establishing prenatal care and third trimester laboratory studies. Unadjusted odds ratios (ORs) and adjusted odds ratios (aORs) were calculated, comparing Medicaid insurance with private insurance with respect to odds of these vaccination outcomes. Robust standard errors were calculated for both specifications, and Hosmer-Lemeshow tests were calculated to indicate goodness of model fit. Analyses were conducted with SPSS (version 25; IBM), and a priori alpha levels were set at 0.05.

Approximately one half of women in the randomly selected sample were white (52.5%), a majority were non-Hispanic (88.0%) and Medicaid enrolled (58.9%), and approximately one third were pregnant for the first time (37%) (Table 1). Overall, 76.2% of women initiated prenatal care during the first trimester, 88.5% completed laboratory tests at both initiation of prenatal care and during the third trimester,^†^ and 61.9% had a vaginal delivery; however, these rates significantly varied by insurance type, with lower rates among women with Medicaid.

**TABLE 1:** Characteristics of Medicaid-insured and privately insured pregnant women who received tetanus toxoid, reduced diphtheria toxoid, and acellular pertussis (Tdap) vaccine (N = 341) and influenza vaccine (N = 316)* during pregnancy — University of Florida Health, Gainesville, Florida, 2016–2018

Characteristic	No. (%)		
	Overall (N = 341)	Medicaid-insured (n = 201)	Privately insured (n = 140)
**Maternal age at delivery, mean (SD)**	28.7 (5.5)	27.2 (5.4)^†^	30.9 (5.1)
**Weeks of gestation at delivery, mean (SD)**	38.7 (1.8)	38.5 (2.0)^†^	39.0 (1.6)
**Maternal race**			
White	179 (52.5)	91 (45.3)^†^	88 (62.9)
Black or African American	100 (29.3)	77 (38.3)^†^	23 (16.4)
Other	62 (18.2)	33 (16.4)	29 (20.7)
**Maternal ethnicity**			
Hispanic or Latino	38 (11.1)	25 (12.4)	13 (9.3)
Non-Hispanic or Latino	300 (88.0)	175 (87.1)	125 (89.3)
Unknown	3 (0.9)	1 (0.5)	2 (1.4)
**Parity**			
1	127 (37.2)	67 (33.3)	60 (42.9)
2	111 (32.6)	56 (27.9)^†^	55 (39.3)
≥3	103 (30.2)	78 (38.8)^†^	25 (17.9)
**Prenatal care initiation (trimester)**			
1st	260 (76.2)	130 (64.7)^†^	130 (92.9)
2nd	66 (19.4)	57 (28.3)^†^	9 (6.4)
3rd	15 (4.4)	14 (7.0)^†^	1 (0.7)
**Mode of delivery**			
Standard vaginal delivery	211 (61.9)	124 (61.7)	87 (62.1)
Operational vaginal delivery	12 (3.5)	6 (3.0)	6 (4.3)
Caesarean	118 (34.6)	71 (35.3)	47 (33.6)
**Completion of prenatal laboratory tests**			
Prenatal care initiation laboratory tests	304 (89.1)	168 (83.6)^†^	136 (97.1)
3rd trimester laboratory tests	339 (99.4)	199 (99.0)	140 (100.0)

**Abbreviation:** SD = standard deviation.

* A total of 25 women for whom the vaccine was not indicated because of documented receipt before pregnancy, allergy to a vaccine component, or lack of availability of the vaccine during prenatal care were excluded from the analysis of influenza vaccination, leaving 316 women in the analysis of influenza vaccination.

**^†^** Indicates statistically significant difference (p<0.05) between Medicaid-insured women and privately insured women.

Among 341 women eligible to receive Tdap, 215 (63.1%) received it, including 123 (36.1%) who were vaccinated during pregnancy and 92 (27.0%) who were vaccinated during the immediate postpartum period (Table 2). This varied significantly by insurance type: 96 of 140 (68.6%) women with private insurance and 27 of 201 (13.4%) with Medicaid received Tdap during the recommended time (27–36 weeks’ gestation) during pregnancy (OR = 0.07; 95% CI = 0.04–0.12, p<0.001). Among women who received Tdap, 77 (74.0%) of those with Medicaid and 15 (13.5%) of those with private insurance received the vaccine in the immediate postpartum period (Table 2) (Figure). Overall, 111 (79.3%) women with private insurance and 104 (51.7%) women with Medicaid received Tdap either during pregnancy or the immediate postpartum period (OR = 0.28; 95% CI = 0.17–0.46, p<0.001).

**TABLE 2:** Tetanus toxoid, reduced diphtheria toxoid, and acellular pertussis (Tdap) and influenza vaccination coverage among pregnant women, by insurance type — University of Florida Health, Gainesville, Florida, 2016–2018

Vaccine/Time of receipt	No. (%)			Bivariate analysis*		Multivariate analysis*^,†^	
	Total (N = 341)	Medicaid insurance (n = 201)	Private insurance(n = 140)	OR (95% CI)	p-value	aOR (95% CI)	p-value
**Tdap**							
During pregnancy	123 (36.1)	27 (13.4)	96 (68.6)	0.07 (0.04–0.12)	<0.001	0.09 (0.05–0.17)	<0.001
Overall^§^	215 (63.1)	104 (51.7)	111 (79.3)	0.28 (0.17–0.46)	<0.001	0.30 (0.17–0.53)	<0.001
**Influenza** ^¶^							
During pregnancy	156 (49.4)	68 (35.6)	88 (70.4)	0.23 (0.14–0.38)	<0.001	0.30 (0.17–0.54)	<0.007
Overall^§^	173 (54.8)	83 (43.5)	90 (72.0)	0.30 (0.18–0.49)	<0.001	0.38 (0.22–0.67)	<0.001

**Abbreviations**: aOR = adjusted odds ratio; CI = confidence interval; OR = unadjusted odds ratio.

* Reference group = private insurance.

^†^ Multivariate analyses adjusted for race, age, parity, gestational age at delivery, trimester of prenatal care initiation, and completion of recommended prenatal initiation studies and third trimester laboratory studies.

^§ ^Receipt during pregnancy or immediate postpartum period.

^¶ ^Ten Medicaid-insured women and 15 privately insured women were excluded from analyses of influenza vaccination because of documentation of receipt before pregnancy, allergy to vaccine components, or lack of availability of the vaccine during prenatal care, leaving an influenza sample of 316.

**FIGURE:**
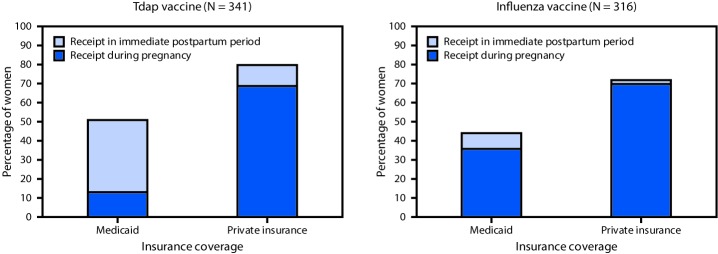
Percentage of pregnant women receiving tetanus toxoid, reduced diphtheria toxoid, and acellular pertussis (Tdap) vaccination and influenza vaccination, by insurance type and timing of receipt relative to pregnancy — University of Florida Health, 2016–2018

Influenza vaccine was received by 54.8% of 316 vaccine-eligible women, including 49.4% who received the vaccine during pregnancy and 5.4% who received it during the immediate postpartum period. Overall, 88 of 125 (70.4%) women with private insurance and 68 of 191 (35.6%) women with Medicaid received influenza vaccine during pregnancy (OR = 0.23; 95% CI = 0.14–0.38, p<0.001); overall, 90 (72.0%) women with private insurance and 83 (43.5%) with Medicaid received influenza vaccine during pregnancy or the immediate postpartum period (OR = 0.30; 95% CI = 0.18–0.49, p<0.001).

Adjusting for patient demographic and prenatal care characteristics did not change these associations. Compared with women who had private insurance, the odds of receiving Tdap during pregnancy were significantly lower among those with Medicaid (aOR = 0.09; 95% CI = 0.05–0.17, p<0.001) (Table 2). Similarly, the odds of receiving influenza vaccine during pregnancy were significantly lower among women with Medicaid than among those with private insurance (aOR = 0.30; 95% CI = 0.17–0.54, p = 0.007). Hosmer-Lemeshow tests indicated that the data were consistent with the assumed model (all p-values >0.10) for all model specifications.

##  Discussion

In a random sample of 341 mothers who delivered at a large, quaternary care and referral academic health center in Florida during 2016–2018, a significantly smaller percentage of Medicaid-insured women received Tdap and influenza vaccines during pregnancy than did privately insured women. This finding is consistent with previous studies demonstrating lower vaccination rates among Medicaid-insured pregnant women (*7*,*8*). However, few studies have included information on receipt of Tdap and influenza vaccines during the postpartum period. Results from this analysis show that compared with privately insured pregnant women, a significantly larger proportion of pregnant women with Medicaid received Tdap and influenza vaccines during the immediate postpartum period, a strategy that confers less protection for infants (*6*).

Under Florida Medicaid guidelines in place during 2016–2018, vaccines, including Tdap and influenza, were not included in the covered pregnancy-related services for pregnant women aged ≥18 years, although Tdap and influenza vaccines were administered in this hospital system in the immediate postpartum period at no additional cost to Medicaid patients. Approximately three fourths of Medicaid-insured women in this study who received Tdap were vaccinated during the immediate postpartum period, suggesting that Medicaid-insured women might receive the Tdap and influenza vaccines as recommended during pregnancy if cost barriers were removed. Florida Medicaid’s lack of coverage for recommended immunizations during pregnancy might have contributed to the lower vaccination rates among Medicaid-insured pregnant women in this study.

The findings in this report are subject to at least four limitations. First, the analyses are limited by the accuracy of the vaccination records available in the patient electronic health records; a vaccine administered at an outside site might not be documented. Second, there is likely to be variation in the number of times a patient was offered these vaccines depending on provider preference and the number of prenatal visits completed (*5*). Third, although the analysis estimated the number of women who would be responsive to vaccination if financial barriers were removed, other factors might have contributed to this decision. Finally, this study was performed at a single university medical center in Florida and might not be generalizable to other settings or states.

In Florida and other states with traditional Medicaid coverage, each state Medicaid program determines whether maternal vaccinations are provided to pregnant mothers with or without cost sharing.^§^ In Florida, Medicaid-insured pregnant women are currently asked to pay for these services themselves or are referred to distant off-site health departments to receive these vaccines on a sliding fee scale. Since the conclusion of this study, Florida announced that as of February 2019 “for enrollees 21 years of age and older (including pregnant women), all (Medicaid) plans elected to cover the influenza vaccine as an expanded benefit.” Removing cost and access barriers that Medicaid-insured women face might increase maternal vaccination coverage in the Medicaid population (*9*).

**Summary**

**What is already known about this topic?**

Vaccination with influenza and tetanus toxoid, reduced diphtheria toxoid, and acellular pertussis (Tdap) vaccines during pregnancy can decrease the risk for influenza and pertussis-associated complications among women and infants, yet vaccination rates remain low. Before 2019, Florida’s Medicaid-covered pregnancy-related services did not include these vaccines; one hospital system covered these vaccines in the immediate postpartum period.

**What is added by this report?**

Among pregnant women who delivered at a Florida health system during 2016–2018, fewer Medicaid-insured than privately insured women received Tdap and influenza vaccines during pregnancy; many women chose to receive vaccination immediately postpartum when provided for free.

**What are the implications for public health practice?**

Medicaid benefits for Tdap and influenza vaccination during pregnancy might increase vaccination coverage.

## Footnotes

* All women who received Tdap during pregnancy were included, including eight who received Tdap outside of the recommended gestational age of 27–36 weeks.

† Laboratory tests at initiation of prenatal care included complete blood count (CBC), urinalysis (UA), and screening for syphilis, rubella, human immunodeficiency virus (HIV), hepatitis B, gonorrhea, and chlamydia. Laboratories during the third trimester included CBC, UA, glucose tolerance test, and screening for syphilis, rubella, HIV, and hepatitis B.

§ https://www.cms.gov/Research-Statistics-Data-and-Systems/Statistics-Trends-and-Reports/MedicareProgramRatesStats/Downloads/MedicareMedicaidSummaries2018.pdf.
